# A Novel Space Robot with Triple Cable-Driven Continuum Arms for Space Grasping

**DOI:** 10.3390/mi14020416

**Published:** 2023-02-10

**Authors:** Yicheng Dai, Zuan Li, Xinjie Chen, Xin Wang, Han Yuan

**Affiliations:** 1 School of Mechanical Engineering and Automation, Harbin Institute of Technology Shenzhen, Shenzhen 518055, China; 2 Guangdong Key Laboratory of Intelligent Morphing Mechanisms and Adaptive Robotics, Shenzhen 518055, China

**Keywords:** cable-driven continuum arms, multi-arm space robot, grasp

## Abstract

With the increasing demand of human beings for space exploration, space robots show great development potential. When grasping space objects with different sizes and shapes, cable-driven continuum arms have better performance than traditional robots. In this paper, a novel space robot with triple cable-driven continuum arms is proposed, which can achieve compliant grasping through multi-arm cooperation. The kinematic model of the robot is proposed and verified through simulations and experiments. Results show that the maximum repeat positioning error is no larger than 1 mm and the maximum tracking error is no larger than 2 mm, compared to the 300 mm long arm. In addition, the demonstration experiment of grasping a ball indicates the good performance of the robot in compliant grasping.

## 1. Introduction

With the rapid development of science and technology, more and more investigations into space are being carried out. Compared to the ways of out-of-cabin operation and maintenance by astronauts, space robots are safer and more flexible [1]. This kind of robot is essential for space activities such as lunar/planetary exploration and in-orbit satellite servicing such as inspection or repairing equipment in space [2].

Benefiting from the large-scale and mature application of joint-link rigid manipulators in industry, traditional space robots mostly use single or multiple traditional arms with rigid joints to operate [3]. However, their rigid link structure tends to be the cause of unwanted collisions which do harm to targets [4,5,6]. Besides, rigid manipulators composed of discrete joints have few degrees of freedom (DOF). Their limitations in dexterity, flexibility and obstacle avoidance ability also make this kind of rigid robot problematic when it comes to meeting the requirements of complex tasks [7,8]. However, due to the explosion of the actuators, encoders, and other electrical components within space atmospheres, the traditional manipulators need much protection which leads to a heavier weight and higher costs to launch. In addition, rigid manipulators often use the end effector to achieve grasping [9]. Usually, there is a custom handle on the target. But when it is applied to non-cooperative targets with unknown shapes and structural size [10], the operation process is complicated and the reliability is poor. All these shortcomings have led us to consider the idea of applying the continuum arms to space robots.

Cable-driven continuum arms are the functional imitation and electronic mechanization of soft animals in the biological world [11], such as snakes [12], cephalopods [13], and elephant trunks [14]. They have great potential in minimally invasive surgeries [15,16], aerospace [7,17], and maintenance in confined spaces [18,19]. Continuum arms have infinite DOF [20]. Therefore, they have much more dexterity and flexibility. They can not only install graspers at the end, but also can achieve whole-arm grasping by twining [13,21], as seen with the elephant trunk or octopus tentacles. This method of grasping is compliant to the shape of the target. There is some research about compliance grasping. However, most of them are about soft grippers [22,23,24]. Yet grippers can only grasp objects of a size no larger than the grippers themselves. As for grasping by using cable-driven continuum manipulators, the size of the objects can be as large as the length of the arm. Moreover, the continuum arms are driven by light cables, which give them the characteristics of electromechanical separation. It is possible for the robot to centrally install parts with electronic components inside the control box to avoid damage from the harsh space environment. Furthermore, this structure is conducive to making the arm lightweight and with low motion inertia, so that the motion precision can be improved. Compared with robots with a single arm, the increase in the number of arms can improve the diversity and reliability of space robot operations [1].

**Figure 1:**
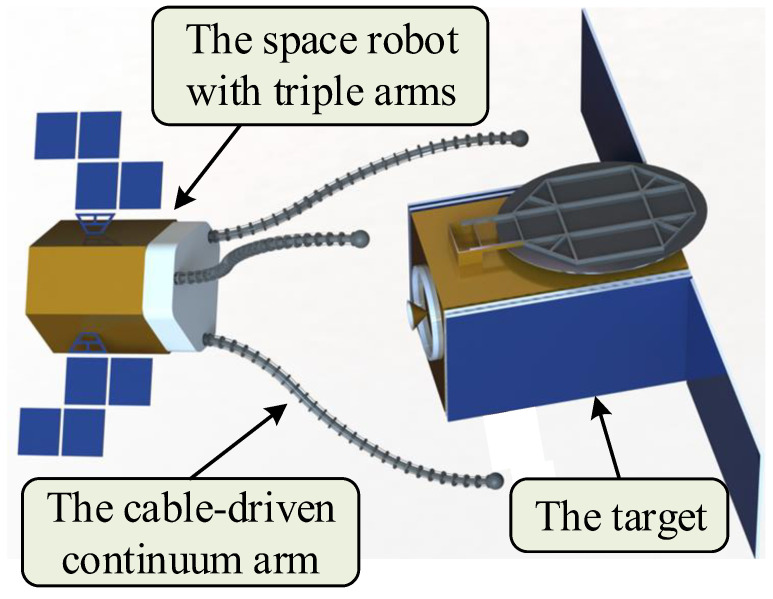
The concept of grasping through space robot with triple arms.

However, there are few studies on continuum arms in the field of space, especially the multi-arm space robot. In this paper, a novel space robot with triple continuum arms is proposed, aiming at compliant grasping and multi-arm cooperative working in space. The concept is shown in Figure 1. To achieve reliable control, the kinematic model of the multi-arm space robot is established. Moreover, simulations of the single-arm motion and multi-arm cooperative grasping are carried out. To verify the performance of the robot in space grasping, a scaled prototype was manufactured and tested. The experiment results indicate that the proposed multi-arm space robot has good performance in collaborative work and compliant grasping.

The rest of this paper is organized as follows. In Section 2, the structure design and the manufacturing method of the proposed multi-arm space robot are introduced. In Section 3, the kinematics model is derived. In Section 4, the trajectory following simulations are carried out and the cooperative grasping strategy is introduced. In Section 5, experimental verifications are conducted, and the results are analyzed. Finally, conclusions are made in Section 6.

## 2. Mechanical Design

For space robots working in space, their structural design needs to consider more issues than conventional robots on the ground. Space robots need to have as many degrees of freedom as possible. The hyper-redundancy makes the robots have superior dexterity. Therefore, they can be used to perform complex tasks in unstructured environments, such as inspection, maintenance, repairing, rescuing, grasping, and so on [10]. In addition, due to the special working environment in aerospace, the exposure of electronic components must be avoided. To achieve this, the robot needs to be divided into two parts: the operating arm and the control box. The electronic equipment is integrated inside the control box where radiation protection and temperature control are used. The operating arm is mainly composed of a central backbone, support disks, and driving cables. The driving cables pass through the cable hole on each support disk and is fixed on the last disk of each section. The motion control of the arm can be realized by driving the cables.

**Figure 2:**
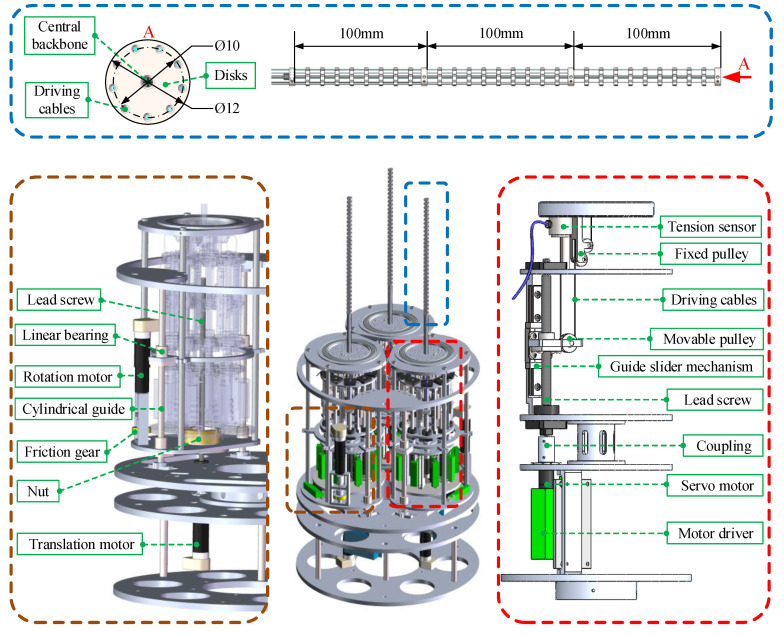
The schematic of the overall structure of the proposed multi-arm space robot.

In this paper, a scaled multi-arm space robot is designed and manufactured. As shown in Figure 2, there are three arms in this prototype. Each arm has three sections, and each section is 100 mm long and driven by three cables which are evenly separated by 120° intervals. There are 10 disks on each section. The diameter of the disk is 12 mm and the diameter of the distribution circle where the cable holes exist is 10 mm.

The central backbone of the arm is made of super-elastic nickel–titanium alloy material. Most support disks are manufactured using 3D printing and glued to the central backbone. The disks at the end of each section are made of steel and fixed by tightening screws for it needs to bear a large tensile force during bending. The above design not only ensures the structural strength of the arm but also reduces its mass, which is about 30 g.

**Figure 3:**
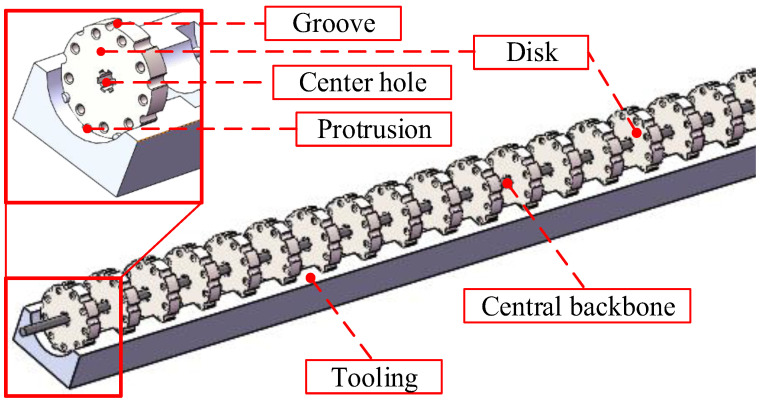
The schematic of the tooling.

To improve the installation accuracy, special tooling is designed as shown in Figure 3. Several semicircular grooves are distributed onto the tooling at equal intervals along the axial direction. When installing, put the disks into the grooves to realize the axial equidistant installation of the disks. The circumferential positioning of the disks is realized via the protrusions and grooves. This ensures that the cable hole on the spaces has a high degree of co-axiality so as reduce the friction between the driving cables and the cable holes.

The control box of each arm of the multi-arm space robot has the same structure. Different from the traditional manipulator with rigid joints, the continuum arm transmits the driving force from the motor to the arm through driving cables. The transmission mechanism of the control box is composed of lead screws, guide slider mechanisms, pulleys, motors, and driving cables. The schematic diagram is shown in the bottom right of Figure 2. After the driving cable bypasses the pulley block, one end is fixed on the tension sensor, and the other end passes through the cable holes on the disks and is connected with the last disk of the corresponding section. The tension sensor can measure the force value of the cable and keep it tensioned. The pulley block can increase the stroke of the driving cable and lower the height of the control box. For a single operating arm in this paper, nine sets of driving components and transmission mechanisms are evenly arranged on the circumference with the operating arm as the center.

Finally, to integrate three continuum arms together, it is necessary to design the integrated control box of the robot. In this paper, the integrated control box can realize the rotation and translation movement of each single control box so as to increase the flexibility of the robot. A lead screw is used to realize the translational movement of the single-arm control box. As shown in the bottom left of Figure 2, the bottom plate of the control box is fixed on the nut of the lead screw. The support is realized via a pair of cylindrical guide rails and linear bearings. This mechanism is driven via the translation motor. As for the rotation movement, a friction gear mechanism is used. It is driven via another motor.

## 3. Kinematic Modeling

To control the behavior of the space robot, the kinematic model must be established. Compared with traditional rigid manipulators, the kinematic model of the continuum arm is much more complex. In this section, the multiple mapping relationship, including the mapping between the joint space and the task space and the mapping between the joint space and the cable space, will be described.

### 3.1. Mapping between Joint Space and Task Space

Due to the flexible structure, the commonly used Denavit–Hartenberg method [25] is not suitable for establishing the kinematics of the continuum arm. Instead, the piecewise constant curvature method [26,27] will be used. 

Nevertheless, the continuum arm still needs to establish a local coordinate system for each section like the rigid arms do. The transformation relationship between the virtual joint angle and the end pose of the arm will be described through homogeneous coordinate transformation. Therefore, it is necessary to establish a complete spatial coordinate system for the proposed multi-arm space robot.

**Figure 4:**
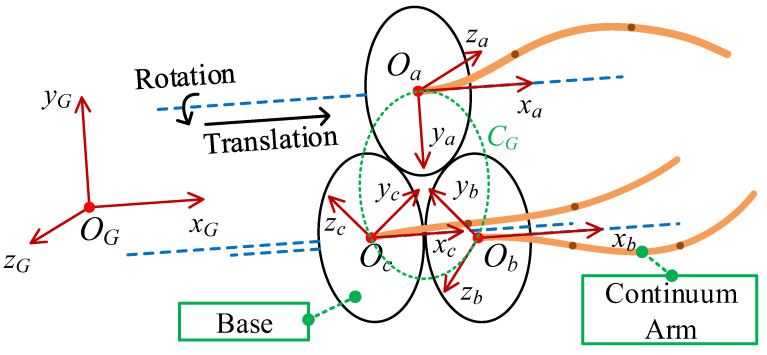
Coordinate system relationship of the space robot.

Firstly, the global coordinate system of the space robot should be established. As shown in Figure 4, the three continuous arms are evenly distributed on the circle $C_{G}$ and centered at $O_{G}$, where the global coordinate system $R_{G}\left\{O_{G},x_{G},y_{G},z_{G}\right\}$ is established. The base coordinate system of each arm is established at $R_{a}\left\{O_{a},x_{a},y_{a},z_{a}\right\}$, $R_{b}\left\{O_{b},x_{b},y_{b},z_{b}\right\}$, and $R_{c}\left\{O_{c},x_{c},y_{c},z_{c}\right\}$, respectively. The y axes of these base coordinate systems all point to $O_{G}$. The x-axes are all perpendicular to the circle and the z-axis is tangent to the circle $C_{G}$ and points to the clockwise direction of $C_{G}$.

**Figure 5:**
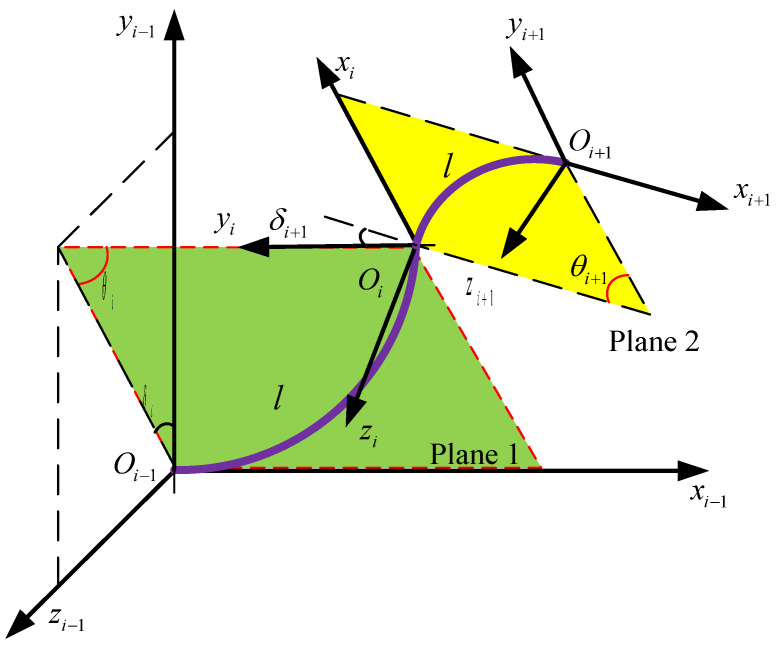
Schematic of the local coordinate system of continuum arm.

Then the local coordinate system on each continuum arm will be established. Supposing the number of the section of each arm is q, then the local coordinate system $R_{i}\left\{O_{i},x_{i},y_{i},z_{i}\right\}$ (0≤i≤q) is shown in Figure 5. Each section of the arm is assumed to be a constant curvature curve [28,29]. The are six DOFs for each arm.

The profile of the $i^{\mathrm{th}}$ section can be described using $\theta_{i}$ and $\delta_{i}$. $\theta_{i}$ is the bending angle of the $i^{\mathrm{th}}$ section and $\delta_{i}$ is the rotating angle of the bending plane with respect to the x-axis of coordinate system $R_{i-1}$. Therefore, six angles $\left\{\theta_{1},\theta_{2},\theta_{3},\delta_{1},\delta_{2},\delta_{3}\right\}$ can be used to describe the shape of each arm.

#### 3.1.1. From Joint Space to Task Space

Based on the above definition, the homogeneous transformation matrix between local coordinate systems $R_{i}$ and $R_{i-1}$ can be obtained according to the following two steps:

(1) Rotate the coordinate system $R_{i-1}$ by angle $\delta_{i}$ around the $x_{i-1}$-axis, and obtain the coordinate $R^{\prime }$.
(2) Rotate the coordinate $R^{\prime }$ by angle $\theta_{i}$ around the $z^{\prime }$-axis. Then translate the origin $O^{\prime }$ to coincide with the origin $O_{i}$ of coordinate $R_{i}$.

Then the homogeneous transformation matrix can be written as:

(1) Tii−1=Tii−1rotate⋅Tii−1bending=[R(xi−1,δi)001]⋅[R(z′,θi)Pi01]

**Figure 6:**
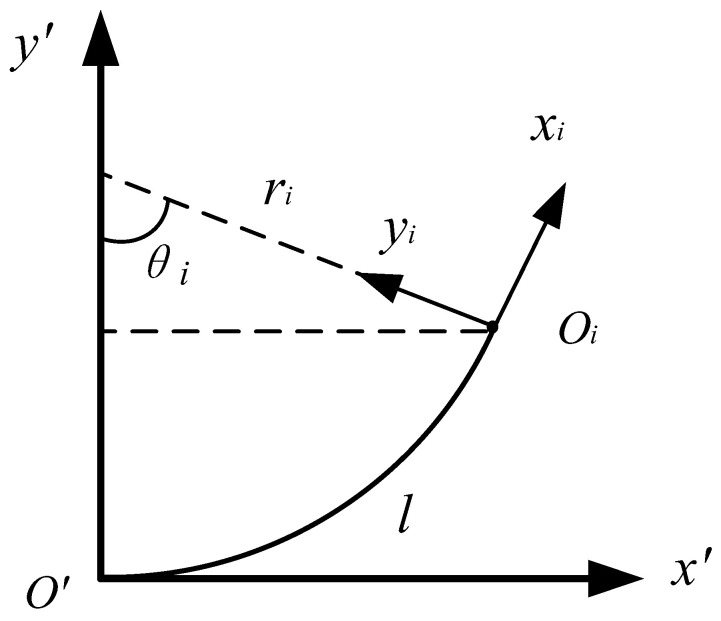
The schematic of a single section.

In Equation (1), the translation vector $P_{i}={\left[x_{O_{i}}^{R^{\prime }},y_{O_{i}}^{R^{\prime }},0,1\right]}^{T}$. As shown in Figure 6, $x_{O_{i}}^{R^{\prime }}$ and $y_{O_{i}}^{R^{\prime }}$ are the expression of the origin $O_{i}$ of coordinate $R_{i}$ in coordinate $R^{\prime }$.

(2) $$x_{O_{i}}^{R^{\prime }}=r_{i}\cdot \sin\theta_{i}$$

(3) $$y_{O_{i}}^{R^{\prime }}=r_{i}(1-\cos\theta_{i})$$

In addition, the multi-arm space robot also includes rotation and translation freedom due to the special integrated control box. Therefore, the homogeneous transformation matrix between the coordinate system of the single arm and the global coordinate system of the arm can be written as:

(4) T0B=[R(xB,α)PB01]

where α is the rotating angle of the single arm. $P_{B}={\left[h,0,0,1\right]}^{T}$ and h is the translation distance. According to Equation (4), we can obtain the transformation matrix from the end coordinate system of the arm $R_{3}$ to the base coordinate system $R_{B}$:

(5) T3B=T0B⋅T10⋅T21⋅T32=f(θ1,θ2,θ3,δ1,δ2,δ3,α,h)

Based on the kinematics of the single continuum arm, we can get the kinematics of the triple-arm space robot. As we can see in Figure 3, the transformation matrix from the $k^{\mathrm{th}}$ arm’s base coordinate system $R_{Bk}$ to global coordinate system $R_{G}$ can be obtained:

(6) $${}_{Bk}^{G}T=\left[\begin{array}{cc} R(x_{G},\varphi_{k}) & P_{G}\\ 0 & 1 \end{array}\right]$$

where k=a,b,c and $\varphi_{k}$ is the rotating angle of the y-axis of global coordinate system $R_{G}$ with respect to the y-axis of base coordinate system $R_{B}$.

(7) T3kG=TBkG⋅T0kBk⋅T1k0k⋅T2k1k⋅T3k2k

Finally, the tip end of the $k^{\mathrm{th}}$ arm PR3kok in global coordinate system $R_{G}$ can be written as:

(8) PRGok=T3kG⋅PR3kok

#### 3.1.2. From Task Space to Joint Space

The control of the robot is task-oriented, which means the joint parameters of the robot are calculated according to the planed pose of the robot end. Therefore, it is necessary to analyze the inverse kinematics of the robot. Different from the forward kinematics solution, the inverse kinematics solution of the robot is related to the joint degrees of freedom $m_{\mathrm{DOF}}$ and the terminal degrees of freedom $n_{\mathrm{DOF}}$ of the robot. In the case of a certain end pose:

- When $m_{\mathrm{DOF}}$ < $n_{\mathrm{DOF}}$, the number of unknowns is smaller than the number of equations. There is no accurate solution.
- When $m_{\mathrm{DOF}}$ = $n_{\mathrm{DOF}}$, the number of unknowns is equal to the number of equations. There is a unique solution.
- When $m_{DOF}$ > $n_{\mathrm{DOF}}$, the number of unknowns is larger than the number of equations. There are infinite solutions.

Normally, the continuum arm is redundant, which means that the number of joint DOFs is larger than the number of end DOFs. As for the space robot with triple arms, the end pose of the arm PRGok is the function of arm shape parameters $\left\{\theta_{1k},\theta_{2k},\theta_{3k},\delta_{1k},\delta_{2k},\delta_{3k},\alpha_{k},h_{k}\right\}$. It can be written as:

(9) PRGok=f(θ1k,θ2k,θ3k,δ1k,δ2k,δ3k,αk,hk)

Inverse kinematics is the process of finding the joint angles while the end pose is known. Usually, optimization functions such as the minimum norm method will be used to obtain the desired set of solutions based on specific objectives and constraints. Otherwise, the linear relationship between the joint angle velocity and the tip velocity can be obtained through the velocity Jacobian. Then the pseudo-inverse solution or an optimal solution under a specific objective can be obtained.

### 3.2. Mapping between Joint Space and Cable Space

The movement of the arm is realized via the bending of sections which are driven by cables. In the above subsection, the shape parameters can be obtained after knowing the end pose of the arm. In this part, the mapping from the joint space to the cable space will be established. The length change in the driving cables can be obtained after knowing the joint angles.

**Figure 7:**
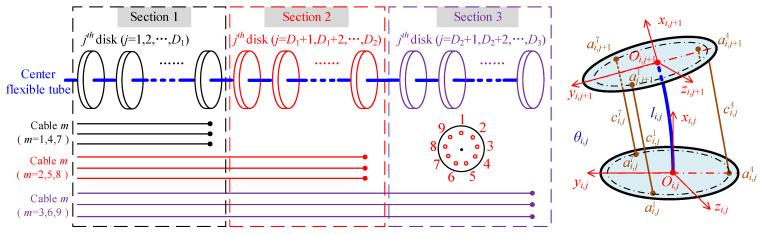
Geometric relationship of the *j*^th^ segment on the *i*^th^ section.

The schematic of a single segment is shown on the right side of Figure 7. $a_{i,j}$ and $a_{i,j+1}$ are cable holes on the $j^{\mathrm{th}}$ and $j+1^{\mathrm{th}}$ disks of the $i^{\mathrm{th}}$ section. $c_{i,j}$ represents the cable length.

Supposing the bending angle of the $i^{\mathrm{th}}$ section is $\theta_{i}$ and the angle of each segment on this section is $\theta_{i,j}$. Then $\theta_{i,j}=\theta_{i}/p$, where p is the number of the segment in each section. Supposing the arc length of the $j^{\mathrm{th}}$ segment is $l_{i,j}$ and the length of the $i^{\mathrm{th}}$ section is $l_{i}$, then $l_{i,j}=l_{i}/p$ (1≤j≤p, 1≤i≤q). In each section, the rotating angle of each segment is equal. The transformation matrix from the $j^{\mathrm{th}}$ disk to the $j+1^{\mathrm{th}}$ disk can be written as:

(10) Ti,j+1i,j=[R(zj,θi,j)pi01]

Then the length of the driving cable of the $j^{\mathrm{th}}$ segment can be written as:

(11) ci,jm=‖ai,jm−Ti,j+1i,j⋅ai,j+1m‖+t

where t is the thickness of disks. In this paper, t=2.5 mm.

Therefore, the whole length of the $m^{\mathrm{th}}$ driving cable $c_{m}$ can be written as:

(12) cm=∑i=1u∑j=1pci,jm=∑i=1u∑j=1p(‖ai,jm−Ti,j+1i,j⋅ai,j+1m‖+t)

where i represents the $i^{\mathrm{th}}$ section and u represents the $u^{\mathrm{th}}$ section that the $m^{\mathrm{th}}$ driving cable passes through, 1≤i≤u≤q. j represents the $j^{\mathrm{th}}$ segment in the $i^{\mathrm{th}}$ section, 1≤j≤p. 

Equation (12) can be used to get the length of driving cable under specific configuration. While the joint angle is obtained, the mapping from joint space to driving cable space can be expressed as:

(13) C=f(θ)

where θ is the vector of joint angles, $\left(\theta =\theta_{1},\theta_{2},\ldots ,\theta_{2q}\right)$. C is the length vector of driving cables. The number of elements in C is 3q. 

It should be noted that if all of the cables are in the position control mode, the continuum arm is redundantly driven, for there are 2q DOFs and 3q cables. Equation (13) is over constrained and there is no accurate solution. However, if there are two cables in the position control mode and one cable is in the force control mode for each section, the number of constraints in Equation (13) is equal to the number of unknowns, meaning there is a unique solution. 

### 3.3. Analysis of Forward and Inverse Kinematics of the Arm

The complex multiple mapping of the cable-driven continuum arm is due to its special driving method which does not control the joints to direct the end pose of the arm, but changes the length of the driving cables instead. 

**Figure 8:**
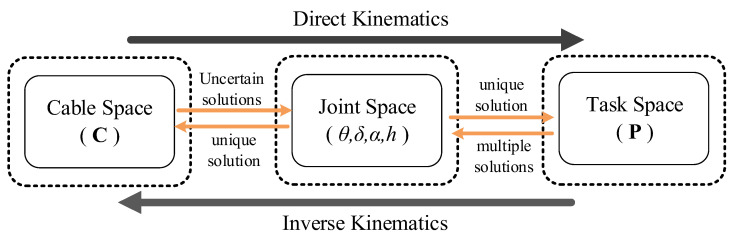
Multilevel kinematic mappings of the cable-driven continuum arm.

As shown in Figure 8, the forward kinematics of the continuum arm is the process of finding the joint angles while the end pose of the arm is known. Since the joint angle is used as the configuration description coordinate, it can be used as an intermediary of coordinate mapping in the forward kinematics model. Firstly, calculate the mapping from the cable space to the joint space, then calculate the joint space to the task space. The inverse kinematics is the opposite process. Normally, continuum arms are redundant, therefore the configuration of the arm corresponding to the same end pose is infinite. However, it is the multi-solution characteristic of inverse kinematics that provides the possibility for configuration optimization to achieve obstacle avoidance, stiffness optimization, etc. As the redundancy increases, the optimization space also expands.

## 4. Simulation

In this section, the simulation analysis of the multi-arm space robot will be carried out in MATLAB, including: the working space analysis of the single arm and multi-arm robot, the trajectory planning of the single arm, and cooperative grasping simulation of the multi-arm robot.

### 4.1. Working Space Analysis

The working space of the robot is the collection of positions and postures that can be reached by the end of the arm. It represents the effective task operation range of the robot. All trajectories must be planned in the working space. In this section, the workspace of the single arm and the multi-arm space robot will be analyzed. 

**Figure 9:**
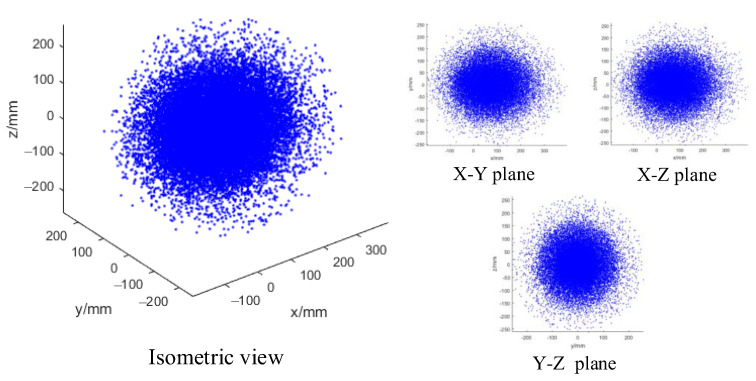
Working space of the single arm.

According to Equation (5), in the base coordinate system $R_{B}$ of the multi-arm space robot, the working space of the robot W can be written as:

(14) $$W=\left\{\begin{array}{c} p_{x}(\theta_{1},\theta_{2},\theta_{3},\delta_{1},\delta_{2},\delta_{3},\alpha ,h)\\ p_{y}(\theta_{1},\theta_{2},\theta_{3},\delta_{1},\delta_{2},\delta_{3},\alpha ,h)\\ p_{z}(\theta_{1},\theta_{2},\theta_{3},\delta_{1},\delta_{2},\delta_{3},\alpha ,h) \end{array}|\begin{array}{c} \theta^{\min}\leq \theta_{i}\leq \theta^{\max},\delta^{\min}\leq \delta_{i}\leq \delta^{\max},i=1,2,3\\ \alpha^{\min}\leq \alpha \leq \alpha^{\max},h^{\min}\leq h\leq h^{\max} \end{array}\right\}$$

where $\left(p_{x},p_{y},p_{z}\right)$ is the end position of the arm in coordinate $R_{B}$. The range of $\left(\theta_{1},\theta_{2},\theta_{3},\delta_{1},\delta_{2},\delta_{3}\right)$ is 0~2π. The rotating angle range of each single arm is 0~2π and the translation range along with x-axis is 0~100 mm. The Monte Carlo principle [30] will be used to analyze the working space of the single arm. As shown in Figure 9, the overall shape of the working space of the single arm is an ellipsoid, and the density of the ellipsoid is uneven. The closer to the interior, the greater the density, which indicates that there are more reachable arm shapes at these points.

**Figure 10:**
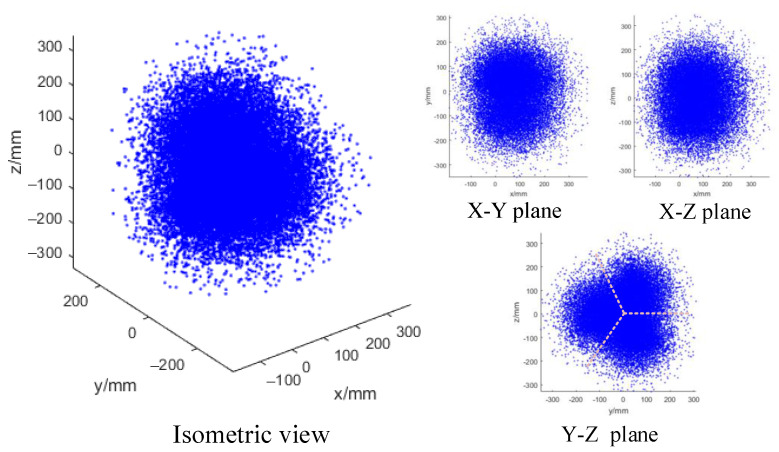
Working space of the triple-arm space robot (the pink dash lines shown in Y–Z plane are to demonstrate the symmetrically distributed working spaces of the three operating arms.).

Similarly, for the space robot integrating three arms, we apply the same method to obtain its workspace. From Equations (7) and (8), the coordinates of the end of the $k^{\mathrm{th}}$ arm in the global coordinate system can be obtained. The working space of the multi-arm space robot is shown in Figure 10. As can be seen in the Y–Z plane view, the working spaces of the three operating arms are symmetrically distributed.

(15) $$W_{k}=\left\{\begin{array}{c} p_{xk}(\theta_{1k},\theta_{2k},\theta_{3k},\delta_{1k},\delta_{2k},\delta_{3k},\alpha_{k},h_{k})\\ p_{yk}(\theta_{1k},\theta_{2k},\theta_{3k},\delta_{1k},\delta_{2k},\delta_{3k},\alpha_{k},h_{k})\\ p_{zk}(\theta_{1k},\theta_{2k},\theta_{3k},\delta_{1k},\delta_{2k},\delta_{3k},\alpha_{k},h_{k}) \end{array}|\begin{array}{c} \theta^{\min}\leq \theta_{ik}\leq \theta^{\max},\delta^{\min}\leq \delta_{ik}\leq \delta^{\max},i=1,2,3\\ \alpha^{\min}\leq \alpha_{k}\leq \alpha^{\max},h^{\min}\leq h_{k}\leq h^{\max},k=a,b,c \end{array}\right\}$$

### 4.2. Trajectory Following Simulation of the Single Arm

To meet the requirements of desired tasks, the arm’s trajectory should be planned first. For the single arm, its structure is relatively simple, and the number of DOF is small. It only needs to realize simple trajectories such as straight lines and arcs. 

When the single arm performs a task, its tip end generally needs to move from the initial straight state to near the object’s position and then adjust the posture of the arm to meet the task requirements. Supposing the end position of the arm in the initial state is $P_{0}$ and the next planned position is $P_{1}$, the position of any point on this straight space line can be written as:

(16) $$P=P_{0}+\varepsilon \frac{\vec{P_{0}P_{1}}}{\left|\vec{P_{0}P_{1}}\right|}(0<\varepsilon <1)$$

where ε is a coefficient. A series of appropriate values can be obtained to get the discrete points of the planned trajectory, and the corresponding joint angle can be obtained through a mapping from the tasking space to the joint space. Then the length of the driving cable can be obtained through a mapping from the joint space to the cable space. 

**Figure 11:**
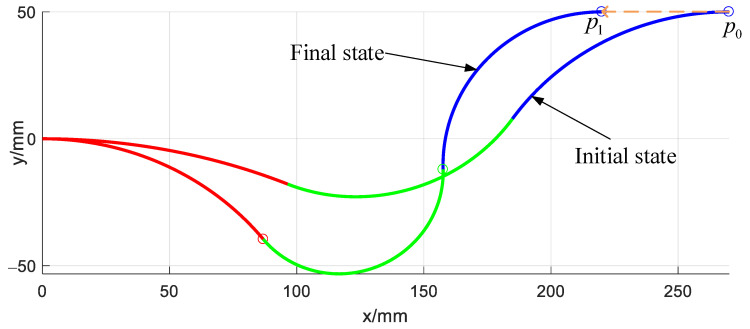
Straight line trajectory following simulation of the single arm.

Figure 11 shows a straight trajectory line of the continuum arm from start point $P_{0}=\left(270,50,0\right)$ to end point $P_{1}=\left(220,50,0\right)$. The end posture of the arm keeps constant during this movement.

**Figure 12:**
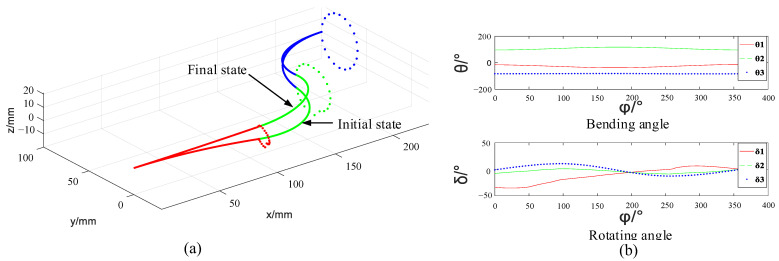
Circle trajectory following simulation of the single arm. (**a**) Circle trajectory following, (**b**) Angle change of the manipulator.

Except for simple lines such as the straight line, the circle line is also common in tasks. The trajectory following simulation in a circle in the Y–Z plane and the corresponding change of the shape angle are shown in Figure 12. The formula of the circle is:

(17) $$\{\begin{array}{l} x=240\\ y=80+20\cos(\varphi )\\ z=20\sin(\varphi ) \end{array}$$

Figure 12a shows that the end pose and the initial pose are the same, but the initial state and the final state can be different. This indicates the multiple solutions of the inverse kinematics. Figure 12b shows that when the arm moves along the planned trajectory, the change of the bending angle and the rotating angle of each section is continuous, which indicates a smooth motion of the arm.

### 4.3. Cooperative Grasping Simulation of the Multi-Arm Space Robot

The main task of the space robot with multi-arm is to grasp the target. To improve the reliability of the grasping process, the robot should have the ability of multi-arm cooperative operation. In this section, a sphere and cylinder are used as the targets and the envelope grasping is realized through the bending of the flexible sections. 

**Figure 13:**
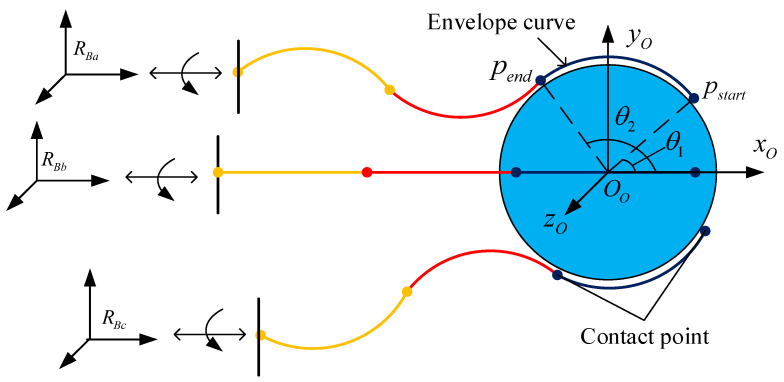
Schematic of the envelope grasping of the multi-arm space robot.

The schematic of the grasping strategy is shown in Figure 13. The deflection radius of the third section of each continuum arm is the same with the target ball. Note the angle between the x-axis of the coordinate system $R_{o}$ and the line connecting the end point $P_{\mathrm{end}}$ of the arm and the target center $O_{O}$, which is $\theta_{1}$. Then the end position $P_{\mathrm{end}}$ and the start position $P_{start}$ of the third section in coordinate $R_{o}$ can be obtained. There are eight DOFs for each arm and two are used for the envelope grasping of the target. 

**Figure 14:**
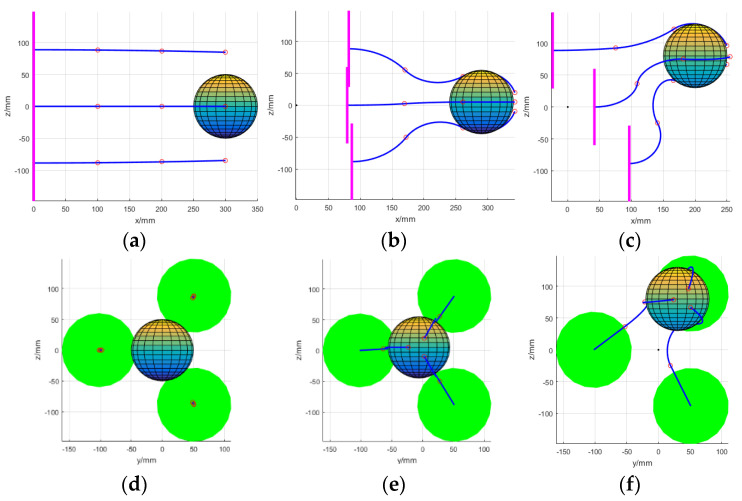
The process of grasping a ball using the multi-arm space robot. (**a**) Front view of initial state, (**b**) front view of middle state, (**c**) front view of final state, (**d**) lateral view of initial state, (**e**) lateral view of middle state, (**f**) lateral view of final state.

For a sphere target, the arm can theoretically perform envelope grasping on any cross-section. However, to ensure the reliability of the grasping, each grasping surface of the arm should pass through the center of the sphere and be evenly distributed. The grasping process is shown in Figure 14.

**Figure 15:**
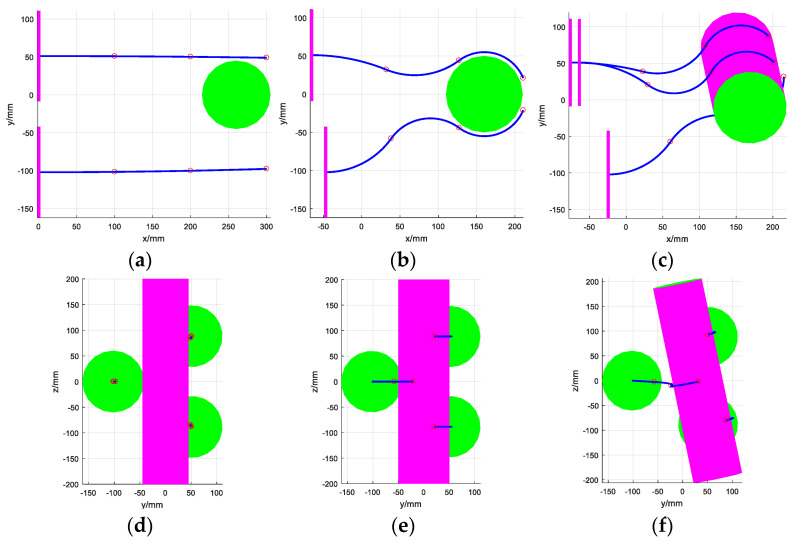
The process of grasping a cylinder using the multi-arm space robot. (**a**) Lateral view of initial state, (**b**) lateral view of middle state, (**c**) lateral view of final state, (**d**) top view of initial state, (**e**) top view of middle state, (**f**) top view of final state.

For a cylinder target, when the grasping surface of each arm is perpendicular to the axis of the cylinder and these three arms are distributed on both sides of the target, the envelope grasping can be realized. After that, translation and rotation movements can be performed to place the target in the desired position. This process is shown in Figure 15.

## 5. Experiment

To verify the kinematics model and motion performance of the robot proposed in this paper, a scale prototype is manufactured, and corresponding experiments are carried out in this part.

### 5.1. Experiments of the Single Arm

**Figure 16:**
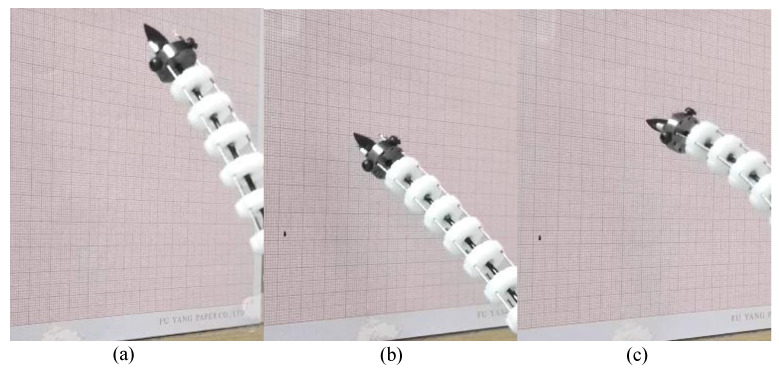
The repeat positioning experiment of the single arm. (**a**) Before marking, (**b**) The 5th marking, (**c**) The 10th marking.

The repeat positioning accuracy experiment is first carried out on the single arm. As shown in Figure 16, a pencil is fixed on the end of the arm. With the movement of the arm, the dots on the graph paper will show the real arriving position of the arm. The initial state of the arm is straight and the distance between the paper and the tip end is 140 mm. A total of 10 experiments were performed and the marked dots are shown in Figure 16. The result shows that all the dots are located within a circle with a diameter of 1 mm.

**Figure 17:**
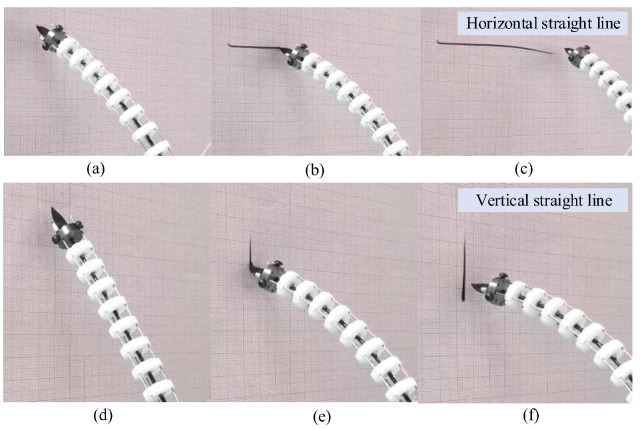
Straight line trajectory following experiments of the single arm. (**a**) Before marking the horizontal line, (**b**) Marking, (**c**) After marking, (**d**) Before marking the vertical line, (**e**) Marking, (**f**) After marking.

Then two groups of trajectory following experiments are carried out to verify the motion performance of the arm. The planned length of the straight line in the horizontal direction is 80 mm and 30 mm in the vertical direction. As we can see in Figure 17a–c, the maximum fluctuation of the tracking trajectory in the horizontal direction is about 2 mm. As for the experiments in the vertical direction shown in Figure 17d–f, the fluctuation of the tracking trajectory is no more than 1 mm. These results indicate that the arm has good performance in trajectory following the straight lines.

**Figure 18:**
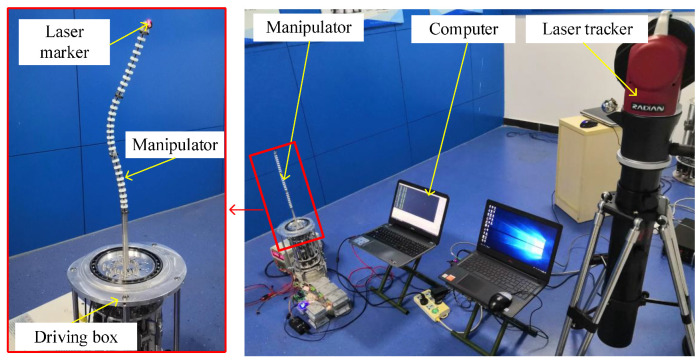
Setup of the circle trajectory following experiment.

**Figure 19:**
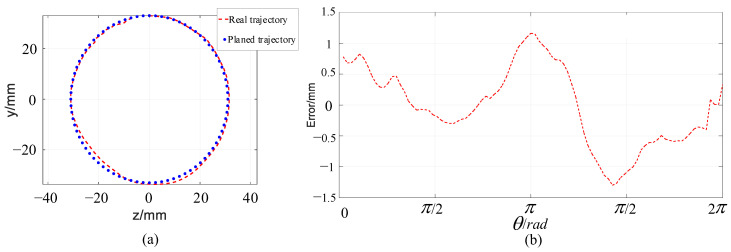
Circle trajectory following experiments of the single arm. (**a**) Trajectory following experiment, (**b**) Trajectory following error.

In this part, the trajectory following experiment in a circle trajectory is carried out. The center of the circle is (270,0,0) and the radius is 30 mm. The tracking trajectory of the arm is measured using a laser tracker, as shown in Figure 18. The error between the tracking trajectory and the planned trajectory is shown in Figure 19. As we can see, the maximum error is no larger than 1.5 mm, which illustrates the good trajectory following performance of the arm.

### 5.2. Cooperative Grasping of the Multi-Arm Space Robot

**Figure 20:**
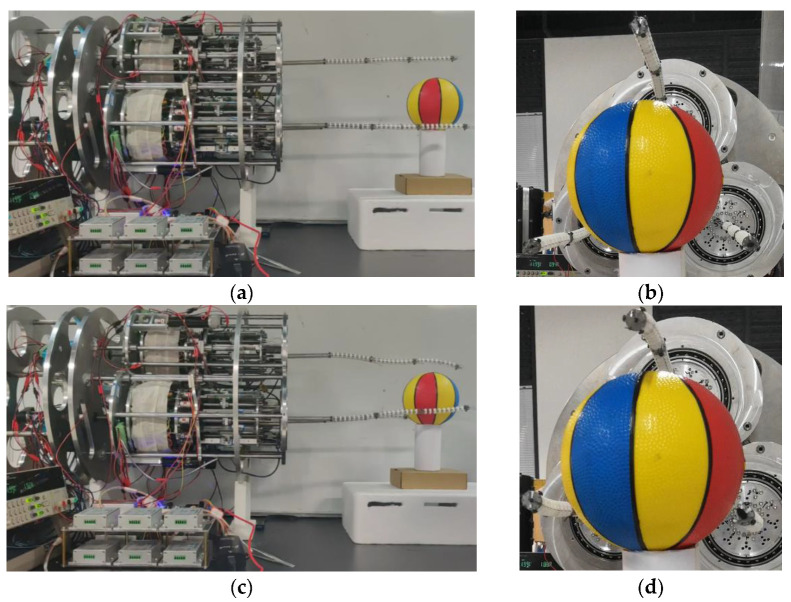

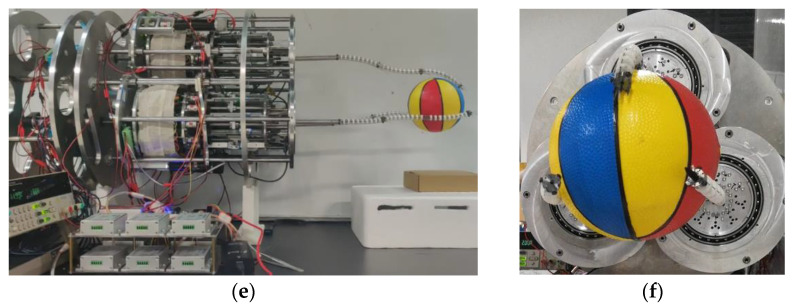
The process of grasping the target ball using the multi-arm space robot. (**a**) Front view of the initial state, (**b**) lateral view, (**c**) front view during grasping, (**d**) lateral view during grasping, (**e**) front view of the final state, (**f**) lateral view of the final state.

After verifying the motion performance and accuracy of the single arm, the cooperative grasping experiment of the proposed space robot with triple arms will be carried out. The target is a ball with a diameter of 100 mm. The process of grasping the target ball is shown in Figure 20. At first, the initial state of these three arms is straight. The target ball is at the center of the three arms. With each arm moving, the ball is finally grasped. This result shows that the multi-arm space robot has the ability of collaborative working and compliant grasping.

### 5.3. Discussion

In this section, the experiments are conducted. The motion performance and the compliance grasping are verified. Although there have been studies on space grasping, their methods are different. For example, in [31], a complicated control algorithm is used to realize soft contact. As for continuum robots with inherent flexible structures, it is not a problem. In [8], a multi-arm space robot integrating both the rigid arm and the cable-driven continuum arm is presented. The rigid arm is responsible for grasping the target and the continuum arm performs on-orbit fine tasks. There exists the same question of soft contacting and compliance grasping. In [32,33], rigid arms with rigid grippers are used to realize approaching and capturing. However, both studies did not realize soft grasping. Therefore, the proposed space robot is important for the realization of compliance grasping tasks in space.

## 6. Conclusions

In order to solve the problem that the traditional robot is not adaptable to grasp space objects with different sizes and shapes, a novel multi-arm space robot with three continuum arms is proposed. The proposed space robot can achieve compliant grasping through multi-arm cooperation. To control the behavior of the space robot, the kinematic model is presented. Moreover, simulations and experimental verifications are carried out to verify the kinematic model and the performance of the robot. According to the results, the maximum repeat positioning error is no larger than 1 mm and the maximum tracking error is no larger than 2 mm in the trajectory following experiments, compared to the 300 mm long arm. In addition, the experiment of grasping a ball indicates the good performance of the robot in compliant grasping and cooperative working.

In the future, more experiments will be carried out to demonstrate the advantage of the novel space robot with multiple continuum arms proposed in this paper. We believe, in the future, this kind of multi-arm robot will play an important role in space development or in any other field where complex operations are required.

## Data Availability

Not applicable.
